# Electrochemical Evaluation of Cd, Cu, and Fe in Different Brands of Craft Beers from Quito, Ecuador

**DOI:** 10.3390/foods12112264

**Published:** 2023-06-04

**Authors:** Oscar López-Balladares, Patricio J. Espinoza-Montero, Lenys Fernández

**Affiliations:** 1 Escuela de Ciencias Químicas, Pontificia Universidad Católica del Ecuador, Quito 170525, Ecuador; lopezoscarl2@hotmail.com (O.L.-B.); lmfernandez@puce.edu.ec (L.F.); 2 Facultad de Ciencias Químicas, Universidad Central del Ecuador, Quito 170521, Ecuador

**Keywords:** analytical chemistry, BDD, craft beer, heavy metals, voltammetry

## Abstract

The presence of heavy metals in craft beers can endanger human health if the total metal content exceeds the exposure limits recommended by sanitary standards; in addition, they can cause damage to the quality of the beer. In this work, the concentration of Cd(II), Cu(II), and Fe(III) was determined in 13 brands of craft beer with the highest consumption in Quito, Ecuador, by differential pulse anodic stripping voltammetry (DPASV), using as boron-doped diamond (BDD) working electrode. The BDD electrode used has favorable morphological and electrochemical properties for the detection of metals such as Cd(II), Cu(II), and Fe(III). A granular morphology with microcrystals with an average size between 300 and 2000 nm could be verified for the BDD electrode using a scanning electron microscope. Double layer capacitance of the BDD electrode was 0.01412 μF cm^−2^, a relatively low value; Ip_ox_/Ip_red_ ratios were 0.99 for the potassium ferro-ferricyanide system in BDD, demonstrating that the redox process is quasi-reversible. The figures of merit for Cd(II), Cu(II), and Fe(III) were; DL of 6.31, 1.76, and 1.72 μg L^−1^; QL of 21.04, 5.87, and 5.72 μg L^−1^, repeatability of 1.06, 2.43, and 1.34%, reproducibility of 1.61, 2.94, and 1.83% and percentage of recovery of 98.18, 91.68, and 91.68%, respectively. It is concluded that the DPASV method on BDD has acceptable precision and accuracy for the quantification of Cd(II), Cu(II), and Fe(III), and it was verified that some beers did not comply with the permissible limits of food standards.

## 1. Introduction

Beer is one of the most consumed alcoholic beverages worldwide, reaching 75% of the market compared to other beverages [1]. In South America, the consumption of craft beer has boomed in recent years due to the constant development and innovation of beers with shades of color in the blonde, red, and black scale, as well as different flavors and alcoholic degrees for a variety of consumers [2,3]. Craft micro-breweries are the main producers of this drink and are the promoters of its great variety in Ecuador. Despite the efforts made by producers to generate beer of higher sensory quality, the hygienic and/or toxicological quality is affected in the production-storage processes, and they do not always follow the same rigorous and automated control of internationally certified industrial macro-breweries. One of the factors that intervene in the quality of both craft and industrial beer is the presence of heavy metals. Heavy metal contamination can come from various sources such as the raw material, barley, the use of additives that contain metallic traces during the fermentation process, the beer maturation (e.g., suspended solids from “green beer” at high temperatures and long times could release more heavy metals into “mature beer”, and contamination from brewing equipment corrosion of the beer [4,5,6].

The presence of heavy metals in the drink may incur non-compliance with food regulations since, due to its toxicity, it can cause lethal effects on the health of consumers [7]. One element is iron (Fe) which, in addition to generating health problems such as hemosiderosis and hemochromatosis [8], also accelerates beer spoilage. Another dangerous contaminant is cadmium (Cd), which can accumulate in the kidneys, where it causes damage to the human filtration mechanism [9]. Another very common metal in drinks is copper (Cu); its long-term consumption can cause stomach aches, vomiting, diarrhea, and liver and kidney damage [10].

In general, the Cd content in alcoholic beverages has been found to be quite low. However, the consumption in large quantities of certain beverages contaminated with this metal can cause significant physiological damage. Side effects include kidney dysfunction, hypertension, liver damage, reproductive toxicity, and bone effects. The kidney is a critical organ for the accumulation of Cd since the half-life of the element in this tissue is about 30 years [11,12].

Some flavor changes appear in beer during aging, which depends on the type of beer and storage conditions; these changes can be caused by the presence of active aromatic carbonyl compounds, which can be formed from radical reactions. Since Fenton’s research was published in 1894, it has been known that Fe ions can catalyze and promote oxidative reactions. In 1934, Haber and Weiss demonstrated that in an aqueous medium, Fe(II) and Cu(II) ions react with H_2_O_2_, generating hydroxyl radicals. It is also known that Fe and Cu ions have a negative influence on the stability of beer flavor; it has even been reported that copper concentrations below 50 µg L^−1^ cause damage to the final product. The origins of these two metals in beer are given from raw materials, brewing equipment, and diatomaceous earth, among others, which have been well investigated [13,14,15].

The levels of heavy metals in beer can also be of agrochemical origin due to residues of insecticides and fungicides, which contain these elements and are used during the cultivation of barley, hops, or other raw material used for the production of beer. It may also be due, among other reasons, to environmental pollution in those places where there are industrial complexes near the grasses [5,16].

Conventional analytical methods used to analyze heavy metals in beverages are flame atomic absorption spectrometry (FAAS), graphite furnace atomic absorption spectrometry (GF-AAS), inductively coupled plasma mass spectrometry (ICP-MS), cold vapor fluorescence atomic spectrometry (CV-AFS), cold vapor atomic absorption spectrometry (CV-AAS), and some chromatographic techniques coupled with spectrometric methods. These methods require adequate treatment of the sample, trained laboratory personnel, very expensive equipment, and considerable response times [17,18,19]. In this sense, electrochemical methods are very accessible for the determination of heavy metals due to their sensitivity, reproducibility, portability, low detection limits of the nanomolar order, and low costs [20,21,22].

Anodic stripping voltammetry (ASV) is a sensitive and reproducible electrochemical technique, which is based on the reduction of a metal M^n+^ to M^0^ on a suitable electrode at a constant reduction potential value for a period; this is the stage of pre-concentration of the analyte of interest. After pre-concentration, the metal on the electrode surface (M^0^) is oxidized to its most stable ion (M^n+^) by means of an oxidation potential sweep, which produces a current signal with an intensity proportional to the concentration of the metal ion present in the dissolution [23,24,25].

In Cuenca-Ecuador, the content of Zn, Cd, Pb, and Cu has been reported in the most consumed craft beers of the blonde, red, and black type, by anodic stripping via polarography, which in total were 24 samples, eight of each type of beer. In the case of Zn, one blonde-type beer exceeded the permitted concentration, according to the INEN 2262 norm of the Ecuadorian Standards Institute (<1 mg L^−1^). In the quantification of Pb, one red type-beer exceeded the allowed concentration (INEN norm establishes < 0.1 mg L^−1^); in the case of Cu, two black beers exceeded the permitted concentration, according to the INEN norm, which establishes < 1 mg L^−1^. On the other hand, the INEN 2262 norm does not regulate the Cd content, which is why, for the analysis of the results obtained from this metal, the Brazilian standard for beers was taken as a reference; all samples were within the allowable limit, <0.5 mg L^−1^ [26]. In addition, the presence of Cu(II), Pb(II), and Cd(II) has been reported in non-alcoholic malt beverages in Iran via ASV. Cu(II), Pb(II), and Cd(II) concentrations of 0.51, 0.04, and 0.05 mg/100 mL, respectively, were detected. According to the acceptable daily intake (ADI) established by the USA, the levels of these metals are below the ADI [27]. Differential pulse adsorption stripping voltammetry (DPAdSV) has also been used to determine Fe in pale, dark, and non-alcoholic lagers. For this purpose, the mercury drop-hanging electrode was used. Free Fe in dark beers was higher than in pale beers, which is still higher than free Fe in non-alcoholic beers; the dark beers presented an average of 121 µg L^−1^ of free Fe, the pale beers 92 µg L^−1^, and the non-alcoholic beers 63 µg L^−1^ [28]. Another study in Quito, Ecuador, to detect Zn and Pb by differential pulse anodic stripping voltammetry (DPASV) in craft beers reported concentrations in the range of 0.0448–0.2740 mg L^−1^ for Pb(II) and 0.3732–0.7056 mg L^−1^ for Zn, of which two of six brands of beers did not comply with the INEN regulations for lead content [29].

The objective of this work was to evaluate Cd, Cu, and Fe in different brands of craft beers from the city of Quito, Ecuador, using DPASV. Boron-doped diamond (BDD) was used as the working electrode, chosen because it is a material with great electrochemical stability against corrosion in aggressive media, a wide working potential window in aqueous and non-aqueous media, low absorption, and low capacitive current, etc. [30,31,32]. Thirteen brands of red beer with the highest consumption were chosen, which are sold at different points of sale in the city. The results obtained from the concentration of the metals analyzed in the different brands of craft beer were verified if they complied with food regulations. The requirements of food regulations are established in Ecuador, in NTE INEN 2262, corresponding to alcoholic beverages. However, it does not contemplate all the metals to be analyzed in this investigation, for which reason the Beer regulations and Brazilian legislation on heavy metals were reviewed, and for fermented alcoholic beverages, regulations applied by Becerra, 2014 [26] were reviewed. These regulations establish the amounts of iron, copper, and cadmium allowed: <0.2 mg L^−1^, <1.0 mg L^−1^ [33], and <0.5 mg L^−1^ [34], respectively.

## 2. Materials and Methods

### 2.1. Materials, Equipment, Reagents, and Samples

#### 2.1.1. Materials and Equipment

For the preparation of solutions, calibrated and amber glassware was used, Nextirrer brand micropipette A ± 5 μL. An electrochemical cell with three heart-type electrodes, an Ag/AgCl reference electrode, a graphite rod counter electrode, and a BDD working electrode (doping level 3000~5000 ppm, 0.3 cm) was used. To weigh the samples, an analytical balance was used, RADWAG model AS 220.R2 A ± 0.0001 g. For electrochemical measurements, a Metrohm Autolab B.V. potentiostat was used. In the treatment of the samples, a heating plate MTOP MS300HS at ± 1 °C was used. In addition, the following was used: SensionTM MM374 potentiometer, BRANSON 1800 brand ultrasound, Thermo Scientific Phenom ProX brand scanning electron microscope (SEM), Perkinelmer AAnalist 400 brand atomic absorption equipment.

#### 2.1.2. Reagents

Cd(II) standard 1000 mg L^−1^, Merck brand; Cu(II) standard 1000 mg L^−1^, Merck brand; Fe(III) standard 1000 μg mL^−1^, AccuStandard brand; H_2_SO_4_ 98% m/m, Merck brand; HNO_3_ 65% m/m, Merck brand; KNO_3_ 99.9% m/m, Fisher brand; acetic acid 99.9% m/m Fisher brand; sodium acetate bran Fisher; H_2_O_2_ 30% *v*/*v*, Fisher brand; N_2_ 99.999% purity; K_3_[Fe(CN)_6_] 99.95% m/m, Fisher Brand; K_4_[Fe(CN)_6_] 99.95% m/m, Fisher Brand; KCl Fisher Brand; trisodium citrate, Fisher Brand, ethylenediamine tetraacetic Acid (EDTA), Fisher brand; NaOH 99.9% m/m, Merck brand, and HCl 37% m/m Merck brand.

#### 2.1.3. Samples

Red beers with alcohol were chosen, and the sampling was carried out in the City of Quito in 13 local breweries in the north-central and northern sectors. A type of random, stratified sampling was applied; for this, different areas of the city of Quito were chosen: center-north in Foch, Orellana, Reina Victoria, Avenue 12 de Octubre, Diego de Almagro, and north of Quito in the Mariana de Jesús, Avenue 6 de Diciembre, Oswaldo Guayasamín, Vaca de Castro, Río Coca, Bicentenario, La Pradera and Whymper, see Table 1. The sample was taken directly from the tap of the drum or barrel (lot number is not included).

The beers were stored in a refrigerator at 5.5 °C until analysis in 500 mL amber glass bottles.

### 2.2. Characterization of the Boron-Doped Diamond Electrode by Scanning Electron Microscope and Electrochemically Using the K_3_[Fe(CN)_6_]/K_4_[Fe(CN)_6_] Redox System

The morphology of the BDD electrode was characterized by SEM, Thermo Scientific Phenom ProX brand; for this, the BDD electrode, clean and dry, was deposited in the sample holder and analyzed at a voltage of 15 kV.

For the electrochemical characterization, a 3-electrode heart cell was used: BDD was used as the working electrode, the Ag/AgCl was used as the reference electrode, and a graphite bar was used as the counter electrode. The electrode was cleaned with 0.2 mol L^−1^ HNO_3_ by cyclic voltammetry (CV) using a Metrohm Autolab B.V. potentiostat. Then a 1 mol L^−1^ KCl solution was prepared and adjusted to pH = 1 with 37% m/m HCl, in which a working window was found by CV, with a scanning speed of 100 mV s^−1^, once the working window was found, 30 cycles were run to condition the electrode before each run, in all cases. 

Double layer capacitance (𝐶_𝑐𝑙_) was then obtained by CV, in 1 mol L^−1^ KCl and pH = 1 (adjusted with HCl as indicated above), in a window from 0.5 V to 1.2 V 6 scan rates were applied: 20, 40, 60, 80, 100 and 120 mV s^−1^. The capacitance was determined by applying Equation (1). Where 𝑣 = sweep speed and *J_p_* is the current density *J_p_* = i/A (i is the current and A is the area of the electrode).

*J_p_* = 𝐶𝑐𝑙 × 𝑣 (1)

Subsequently, BDD’s electrochemical response was studied against the redox couple K_3_[Fe(CN)_6_]/K_4_[Fe(CN)_6_] 4 mmol L^−1^, in KCl 1 mol L^−1^ as electrolyte adjusted to pH = 1 with HCl, and different 𝑣: 20, 40, 60, 80, 100 y 120 mV s^−1^. The Randles-Sevcik equation was applied, which describes the effect of the 𝑣 at maximum response current or peak current I_p_ (I_p_ vs. ν^1/2^), Equation (2), where I_p_: is the maximum current, n: number of electrons transferred in the redox process, A: is the area of the electrode, F: Faraday’s constant, D: diffusion coefficient, C: concentration). In addition, ΔE_p_ = (Ep_ox_ − Ep_red_), half-wave potential (Ep_1/2_ = (Ep_ox_ − Ep_red_)/2), and the ratio (Ip_ox_/Ip_red_) were evaluated. In addition, the electron exchange rate constant was assessed, k° according to the Nicholson equation, Equation (3) (where k°: rate constant of electron exchange, Ψ: function of the heterogeneous rate constant of the electron, D_0_: diffusion constant of the chemical species, F: constant of Faraday, υ: sweep speed, R: universal gas constant, T: temperature (25 °C) [35].

(2) $$I_{p}=2.69\times 10^{5}n^{\frac{3}{2}}{\mathrm{AD}}^{\frac{1}{2}}{C\upsilon }^{\frac{1}{2}}$$

(3) $$k{}^{\circ}=\frac{\Psi {\left({\pi D}_{0}F\upsilon \right)}^{1/2}}{{\left(\mathrm{RT}\right)}^{1/2}}$$

### 2.3. Preparation of the Craft Beer Sampler

Preparation of the beer sample followed the following steps: (i) degassing 25 mL of craft beer by ultrasound (BRANSON 1800 brand) at 30 °C for 20 min; (ii) acid digestion of the degassed craft beer sample with 5 mL of 65% m/m concentrated HNO_3_ and 2 mL of 30% m/m H_2_O_2_. Heating at 100 °C on the MTOP MS300HS heating plate until a yellow coloration is achieved; (iii) the digestion product was measured, with the corresponding support electrolyte solution (the type of electrolyte, the concentration, and the pH depending on the metal to be determined, see Section 2.4.1), up to a final volume of 25 mL. The final pH was controlled by adding 2 mol L^−1^ NaOH and using the SensionTM MM374 pH meter potentiometer; (iv) prior to the measurements, it was purged for 10 min with 99.99% N_2_ to eliminate O_2_ molecules that could cause interference in the analysis.

### 2.4. Procedure for the Evaluation of Cd, Cu, and Fe by Differential Pulse Adsorption Stripping Voltammetry

#### 2.4.1. Selection of Electrolyte Support

Samples of known concentration were prepared from standard solutions of 100 μg L^−1^ of Cd(II), 90 μg L^−1^ of Cu(II), and 80 μg L^−1^ of Fe(III) in several support electrolytes to evaluate the medium that gives the best response.

To evaluate Cd(II), 0.1 mol L^−1^ acetic acid/0.055 mol L^−1^ sodium acetate at pH 4.5 was used as an electrolyte, according to what was reported by Loaiza (2020) [30].

To evaluate Cu(II), the following electrolytes were tested: (i) acetic acid 0.1 mol L^−1^/sodium acetate 0.055 mol L^−1^ at pH 4.5; (ii) KCl 0.1 mol L^−1^/HCl 0.01 mol L^−1^ and (iii) with KNO_3_ 0.1 mol L^−1^/HNO_3_ 0.01 mol L^−1^.

To evaluate Fe(III) were tested: (i) 0.1 mol L^−1^ sodium citrate, (ii) 0.1 mol L^−1^ EDTA and (iii) 0.1 mol L^−1^ KNO_3_. Once the electrolytes with the best responses for each analyte were selected using DPASV, a study of the effect of pH was carried out using different concentrations of acid: in HNO_3_ 0.01 mol L^−1^ (pH = 2.10), HNO_3_ 0.1 mol L^−1^ (pH = 1.20), and no acid (pH = 7.00).

Appropriate electrolytes for Cu(II) and Fe(III) were chosen by DPASV using a Metrohm Autolab B.V potentiostat. 

#### 2.4.2. Determination of Optimal Differential Pulse Adsorption Stripping Voltammetry Parameters

Standard analyte solutions of concentration 100 μg L^−1^ of Cd(II), 90 μg L^−1^ of Cu(II), and 80 μg L^−1^ of Fe (III) were prepared in the medium of selected support electrolyte in the Section 2.4.1. DPASV was applied to each solution in the potential range of −2.4 to 1.0 V. Modulation amplitude (MA), modulation time (MT), and time interval (TI) properties were varied from 0.05 V, 0.05 s, and 0.05 s (respectively) until a well-defined signal is achieved. In addition, the pre-concentration potential and the pre-concentration time of DPASV were evaluated.

#### 2.4.3. Construction of the Calibration Plot and Determination of the Detection and Quantification Limit

Standard solutions of Cd(II), Cu(II), and Fe(III) were prepared; of concentration 40, 80, 120, 160, and 200 μg L^−1^ for Cd(II), and 30, 60, 90, 120, and 150 μg L^−1^ for Cu(II) and Fe(III), for separated, in supporting electrolyte selected in Section 2.4.1. The current intensity signal was measured by DPASV of each solution in the potential range of −2.0 to −0.5 V after measuring the blanks. Then the analyte oxidation peaks were detected, and the calibration plots expressed as current intensity versus analyte concentration in μg L^−1^ were performed. Subsequently, the equation of the plot for each analyte was determined.

Then 10 support electrolyte solutions were prepared, and the current intensity signal was measured by DPASV of each solution at the same potential detected for the current peaks of the calibration plot. Once the readings were obtained, the detection limit and quantification limit (DL and QL) were calculated.

#### 2.4.4. Search for Optimal Differential Pulse Adsorption Stripping Voltammetry Parameters in the Craft Beer Sample

A previously digested and fortified craft beer solution was prepared with the heavy metal standard in a concentration between 80 to 200 μg L^−1^, and they were measured with the corresponding electrolytes. Concentrations for fortification were: 200 μg L^−1^ of Cd(II), 90 μg L^−1^ of Cu(II), and 80 μg L^−1^ of Fe(III).

DPASV was applied, and the working window was found between −2.4 V to 1.0 V. The properties of modulation amplitude, modulation time, and time interval were varied until obtaining a definite and strong current signal.

#### 2.4.5. Standard Addition Plot

Standard addition solutions were prepared with Cd(II), Cu(II), and Fe(III) of concentration 0, 40, 80, 120, 160, and 200 μg L^−1^ of Cd(II) and 0.30, 60, 90, 120, and 150 μg L^−1^ of Cu(II) and Fe(III) separately. The calibration plots were built with the voltammetric signals obtained by DPASV of each solution in the potential range of −2.05 to −0.5 V. From the data obtained, the linear adjustment was made, and with the equation generated, the initial concentration of the craft beer (X*m*) was calculated by extrapolation.

#### 2.4.6. Evaluation of Repeatability, Reproducibility, and Recovery in the Determination of Cd, Cu, and Fe by Differential Pulse Adsorption Stripping Voltammetry

For the determination of repeatability, reproducibility, and recovery percentage, digested and fortified craft beer solutions were evaluated at 3 concentration levels (*μ*): 40, 120, and 200 μg L^−1^ of Cd(II); 30, 90, and 120 μg L^−1^ of Cu(II), and 30, 90, and 120 μg L^−1^ of Fe(III).

To evaluate the repeatability, the samples were run in triplicate by means of DPASV on the same day (total 9 determinations). With the current values obtained and the equations of the linear regressions by standard addition (Section 2.4.5), the concentrations of the sample + added standard (Y*m* + *x*) were evaluated. Subsequently, the standard deviation (S_d_) and the percentage of the relative standard deviation (RSD%) were calculated.

To evaluate the reproducibility, the samples were run in triplicate on 3 different days (total of 27 determinations) using DPASV. To find the sample concentration, *Sd*, and RSD %, the above procedure was followed.

To evaluate the recovery percentage (R%), the concentrations obtained in repeatability and reproducibility ($Y_{m+x}$), and the respective percentages were calculated according to Equation (4), where $Y_{m+x}$ = concentration of the sample + added standard, $X_{m}$ = initial concentration of the sample, μ = concentration of the added standard.

(4) $$R\%=\frac{Y_{m+x}-X_{m}}{\mu }*100$$

### 2.5. Determination of the Concentration of Heavy Metals Cd, Cu, and Fe in Craft Beers Using Differential Pulse Adsorption Stripping Voltammetry

Samples of craft beer (digested) fortified with analyte standards (Section 2.4.5) and measured with the corresponding electrolyte were evaluated in triplicate. DPASV was applied, and the current intensity peaks of each solution were measured. Current intensity peaks (I) of the voltammograms were measured, and by linearization I vs. C, the equation of the plot was determined, which allowed the calculation of the metal concentration in the beer. The process was repeated for the different craft beers (n = 13).

### 2.6. Determination of the Concentration of Heavy Metals Cd, Cu, and Fe in Craft Beers by Flame Atomic Absorption Spectrometry

FAAS was applied to the craft beer samples because it is the most widely used technique for the quantification of heavy metals due to its ability to determine more than 70 elements in solution and in different matrices [36]. The beer samples were prepared in the same way as for the electrochemical method, as described in Section 2.3. Standard addition calibration plots were prepared in the same way as for DPASV. However, other linear ranges were applied due to the difference in sensitivity of the FAAS equipment (Perkinelmer brand AAnalist 400) compared to the DPASV method. Regarding the calibration of Cd(II), a linear range of 0.01 to 1 mg L^−1^ was used; for Cu(II) from 0.05 to 1.5 mg L^−1^ and for Fe(III) from 0.3 to 5 mg L^−1^. The DL values for Cd, Cu, and Fe are 0.0045, 0.0164, and 0.1337 mg L^−1^, respectively; the QL values for Cd, Cu, and Fe are 0.0151, 0.0546, and 0.4457 mg L^−1^, respectively. Cd was measured at a wavelength (λ) of 228.8 nm, Cu at 324.75 nm, and Fe at 302.06 nm, and three measurements of each type of sample were made. Once the readings were obtained, the arithmetic mean, the standard deviation, and the percentage of the real standard deviation were calculated. In addition, Student’s “t” was calculated using SPSS Statistics to compare whether there are significant differences between the two applied methods (DPASV vs. FAAS) for the quantification of the 3 heavy metals (Cd, Cu, and Fe).

## 3. Results

### 3.1. Microscopic Characterization of the Boron-Doped Diamond Electrode

**Figure 1:**
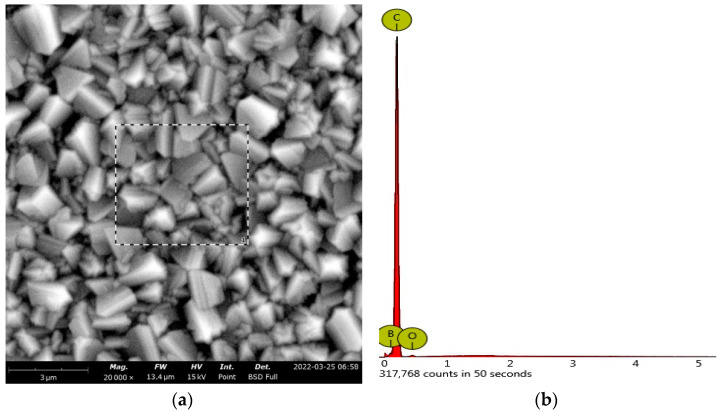
(**a**) SEM micrograph of BDD electrode of size 3 µm and scale 13.4 µm, Mode: 15 kV. (**b**) EDS of the BDD electrode.

Figure 1 shows the granular morphology of the BDD electrode by SEM; it presents microcrystals with an average size between 300 and 2000 nm. A surface free of impurities is observed. The crystals present a relatively uniform distribution; they do not present holes or cracks on their surface; that is, the BDD presents a characteristic micrograph of a clean electrode and is suitable for use in electroanalysis [37]. The study by energy dispersive spectrometer (EDS) of the BDD yields a composition: carbon 94.11% atomic, 94.26% *w/w*; boron 4.91% atomic, 4.43% *w/w*; 0.98% atomic oxygen, 1.31% *w/w*, 0.052 B/C. The B/C value recorded in this study is within the range determined by Xu and Einaga (2020), from 0.03 to 2.20, which is characteristic of boron-doped diamond electrodes [38,39].

### 3.2. Electrochemical Characterization of the Boron-Doped Diamond Electrode

**Figure 2:**
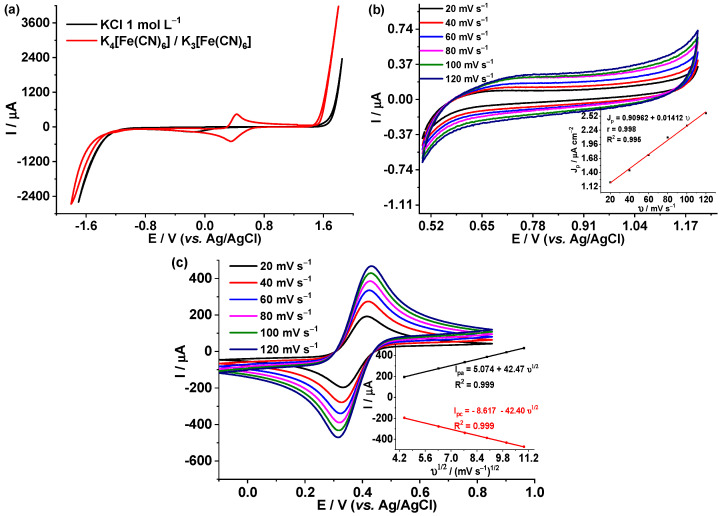
(**a**) Cyclic voltammograms of BDD in 1.0 mol L^−1^ KCl, pH = 1 and 4 mmol L^−1^ K_4_[Fe(CN)_6_]/K_3_[Fe(CN)_6_], 1.0 mol L^−1^ KCl electrolyte, pH = 1, *v* = 100 mV s^−1^. (**b**) Voltamometric response of BDD in KCl 1.0 mol L^−1^ at pH = 1, at different *v*. Insert: I vs. *v.* (**c**) Voltamometric response for the K_4_[Fe(CN)_6_]/K_3_[Fe(CN)_6_] 4 mmol L^−1^ (both redox couples), in KCl 1 mol L^−1^ electrolyte, pH = 1. Insert I_p_ vs. *v*^1/2^.

Figure 2a shows the characteristic electrochemical behavior of the BDD when its surface is clean. A working window potential is observed between −1.15 V and 1.60 V in 1 mol L^−1^ KCl as electrolyte at pH = 1 (adjusted with HCl 1 M); the work window is similar to the report done by Bogdanowicz et al. (2020), −1.5 to 1.5 V [40]. Therefore, in this potential interval, any electroanalytical study can be carried out without interference from the medium [41,42]. In addition, the electrode response was evaluated against the K_4_[Fe(CN)_6_] 4 mmol L^−1^—K_3_[Fe(CN)_6_] 4 mmol L^−1^ system; the oxidation and reduction signals appear at 0.42 and 0.32 V, respectively. These responses are characteristic of BDD when it is clean and ready to be used in electroanalysis [43,44].

Figure 2b shows the electrochemical response of the BDD at different scan rates (*v*) in 1.0 mol L^−1^ KCl at pH = 1, in a potential interval of 0.5 V to 1.2. V. It is observed that as *v* increases, the capacitive current increases. To determine the double layer capacitance, a potential of 1.2 V was set, and a plot was constructed, maximum currents (I_p_) vs. *v* (mV s^−1^), insert in Figure 2b. From the slope of the linear fit, the double layer capacitance (C_dl_) was calculated according to Equation (1). A value of C_dl_ = 0.01412 μF cm^−2^ was obtained; this value corresponds to that reported in the literature for the case of BDD with a low concentration of C-sp^2^ [45]. 

Figure 2c shows the electrochemical response of BDD, at different *v*, against the redox couple K_4_[Fe(CN)_6_]/K_3_[Fe(CN)_6_] in KCl 1 mol L^−1^ pH = 1. In the insert of Figure 2c, the I_p_ increases linearly with υ^1/2^, characteristic behavior of BDD when its surface is clean and there are no diffusional complications. The standard rate constant (k°) was determined with the Nicholson equation [35], Equation (3), using an average value of ΔE of all the scan rates studied. The average value obtained from k° = 2.44 × 10^−2^ ± 4.67 × 10^−3^ cm s^−1^, and it is similar to the values obtained by Rehascek et al. (2020), 1.01 × 10^−2^ to 3.60 × 10^−3^ cm s^−1^ [46]. This indicates that the redox process is fast and quasi-reversible [47,48,49]; see Table S1.

### 3.3. Determination of Cd(II), Cu(II), and Fe(III) by Differential Pulse Adsorption Stripping Voltammetry at Boron-Doped Diamond Electrode 

#### 3.3.1. Selection of Supporting Electrolyte for the Determination of Cd(II), Cu(II), and Fe(III) by Differential Pulse Adsorption Stripping Voltammetry

Evaluation of Cd(II) by DPASV at BDD electrode, the electrolyte acetic acid 0.1 mol L^−1^/sodium acetate at pH 4.5 produces the sharpest current signals and, without interference, agrees with what is reported in the literature by Loaiza, (2020), see Figure 5a–c.

**Figure 3:**
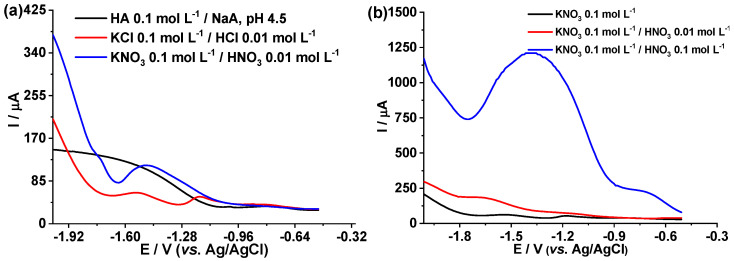
(**a**) DPASV on BDD of 90 μg L^−1^ of Cu(II) in different electrolytes: acetic acid (HA) 0.1 mol L^−1^/sodium acetate (NaA) pH 4.5, KCl 0.1 mol L^−1^/HCl 0.01 mol L^−1^, and KNO_3_ 0.1 mol L^−1^/HNO_3_ 0.01 mol L^−1^; (**b**) DPASV in BDD of 90 μg L^−1^ of Cu(II) in KNO_3_ 0.1 mol L^−1^, KNO_3_ 0.1 mol L^−1^/HNO_3_ 0.01 mol L^−1^, and KNO_3_ 0.1 mol L^−1^/HNO_3_ 0.1 mol L^−1^.

Figure 3a reports the voltammograms generated by DPASV in the BDD electrode for Cu(II) 90 μg L^−1^ in different electrolytes. The parameters were: modulation amplitude (MA) 0.1 V, modulation time (MT) 0.1 s, and time interval (TI) 0.1 s, with pre-concentration of −1.5 V for 1 min. In 0.1 mol L^−1^ acetic acid/sodium acetate pH 4.5 solution, the copper oxidation peak could not be identified. For KCl 0.1 mol L^−1^/HCl 0.01 mol L^−1^ and KNO_3_ 0.1 mol L^−1^/HNO_3_ 0.01 mol L^−1^, the copper current signal appears from −1.3 V to −0.95 V. The KNO_3_ 0.1 mol L^−1^ electrolyte was selected since the Cu(II) oxidation signal is the sharpest and more defined.

Once the KNO_3_ electrolyte was selected, the pH was changed by adding HNO_3_ in order to improve the response current signal in the detection of Cu(II) by DPASV. Figure 3b reports the electrochemical response of Cu(II) 90 μg L^−1^ in: KNO_3_ 0.1 mol L^−1^, KNO_3_ 0.1 mol L^−1^/HNO_3_ 0.01 mol L^−1^ (pH = 2.10) and KNO_3_ 0.1 mol L^−1^/HNO_3_ 0.1 mol L^−1^ (pH = 1.20), with the previously used DPASV parameters. The sharpest current signal between −1.76 V to −0.89 V was obtained in the electrolyte KNO_3_ 0.1 mol L^−1^/HNO_3_ 0.1 mol L^−1^ (pH = 1.20).

**Figure 4:**
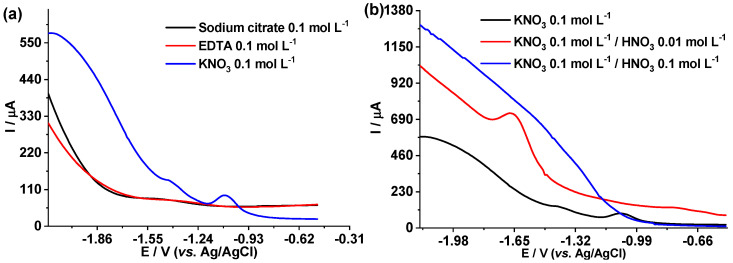
(**a**) DPASV on BDD for 80 μg L^−1^ of Fe(III) in different electrolytes: 0.1 mol L^−1^ sodium citrate, 0.1 mol L^−1^ EDTA, and 0.1 mol L^−1^ KNO_3_; (**b**) DPASV of the BDD for 80 μg L^−1^ of Fe (III) in: KNO_3_ 0.1 mol L^−1^, KNO_3_ 0.1 mol L^−1^/HNO_3_ 0.01 mol L^−1^, and KNO_3_ 0.1 mol L^−1^/HNO_3_ 0.1 mol L^−1^.

Figure 4a shows the voltammograms of 80 μg L^−1^ of Fe(III) in different electrolytes, using DPASV, with the following parameters: MA 0.1 V, MT 0.1 s, TI 0.1 s, pre-concentration of −1.5 V for 1 min. An oxidation signal was achieved in KNO_3_ between 1.20 V and −0.90 V. No signal was achieved in the other electrolytes [50].

In order to improve the Fe(III) signal, different concentrations of HNO_3_ were assessed for the selected electrolyte, KNO_3_. Figure 4b shows the DPASV voltammogramas of 80 μg L^−1^ of Fe(III) in: KNO_3_ 0.1 mol L^−1^, KNO_3_ 0.1 mol L^−1^/HNO_3_ 0.01 mol L^−1^ (pH = 2.10) and KNO_3_ 0.1 mol L^−1^/HNO_3_ 0.1 mol L^−1^ (pH = 1.20) [50,51]. The DPASV parameters were the same used previously. A more defined and more intense current signal was achieved for the case of KNO_3_ 0.1 mol L^−1^/HNO_3_ 0.01 mol L^−1^, between −1.78 V to −1.47 V, so this support electrolyte was selected for the determination of Fe(III).

#### 3.3.2. Optimal Differential Pulse Adsorption Stripping Voltammetry Parameters for the Determination of Cd(II), Cu(II), and Fe(III)

**Figure 5:**
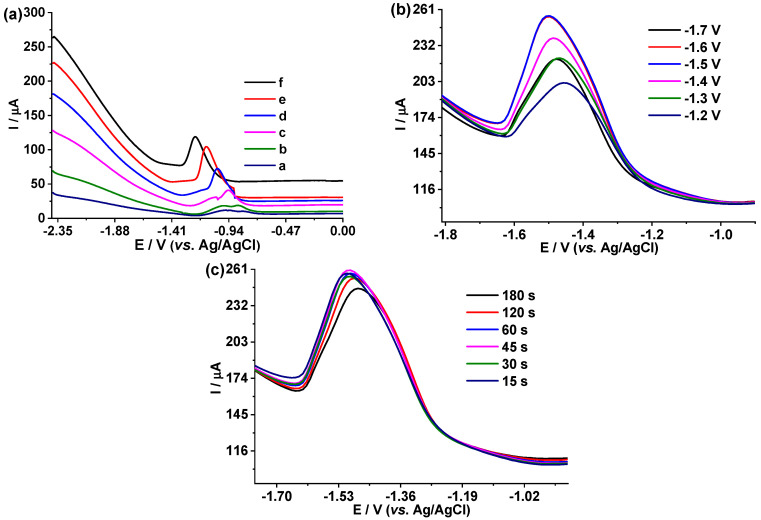
DPASV on BDD of 100 μg L^−1^ of Cd(II) in 0.1 mol L^−1^ acetic acid/sodium acetate, pH 4.5, for different DPASV parameters: (**a**) *a.* MA 0.05 V, MT 0.05 s, TI 0.05 s; *b.* MA 0.1 V, MT 0.1 s, TI 0.1 s; *c.* MA 0.2 V, MT 0.2 s, TI 0.2 s; *d.* MA 0.3 V, MT 0.3 s, TI 0.3 s; *e.* MA 0.4 V, MA 0.4 s, TI 0.4 s; *f.* MA 0.5 V, MT 0.5 s, TI 0.5 s. (**b**) At different pre-concentration voltages: −1.2 V, −1.3 V, −1.4 V, −1.5 V, −1.6 V, −1.7 V. (**c**) At different pre-concentration times: 15 s, 30 s, 45 s, 60 s, 120 s, 180 s.

The parameters of DPASV at the BDD electrode for the quantification of Cd(II) 100 μg L^−1^ of Cd(II) was used in electrolyte support of 0.1 mol L^−1^ acetic acid/sodium acetate pH 4.5. In Figure 5a, a cadmium stripping signal between −1.4 V to −0.7 V is observed. 

In order to improve the Cd(II) response signal, the MA, MT, and TI parameters were varied from 0.05 V, 0.05 s, and 0.05 s to 0.5 V, 0.5 s, and 0.5 s, respectively. When the values of MA, MT, and TI were larger, a stronger and more defined stripping signal was achieved. However, the parameters MA = 0.4 V, MT = 0.4 s, and TI = 0.4 s were chosen for the quantification of Cd(II), lower background current is generated with these parameters.

Figure 5b shows the effect of the pre-concentration potential of Cd(II) in the previously selected support electrolyte, with 100 μg L^−1^ of Cd(II); at higher potential values, current peaks are more intense. However, when −1.6 V and −1.7 V pre-concentration are applied, the responses are similar, so −1.6 V was chosen for the sample reading. Figure 5c shows the effect of the pre-concentration time of Cd(II) 100 μg L^−1^, from 15 s to 180 s in the previously selected electrolyte. Little variation in the intensity of the peak was observed, so 15 s was selected as the pre-concentration time. This time will allow us to have fast measurements, which is what is sought in analytical methods.

Similarly to Cd(II), we proceeded with Cu(II) and Fe(III) in order to find the optimal parameters: 

In the case of Cu(II), 90 μg L^−1^, in the previously selected support electrolyte, KNO_3_ 0.1 mol L^−1^/HNO_3_ 0.1 mol L^−1^, a potential window from −1.7 to −1.0 V was obtained.

In order to improve the Cu(II) response signal, the MA, MT, and TI parameters were varied from 0.05 V, 0.05 s, and 0.05 s to 0.3 V, 0.3 s, and 0.3 s, respectively. When the values of MA, MT, and TI were larger, the sharpest current signal was achieved. However, at values of MA = 0.3 V, MT = 0.3 s, and TI = 0.3 s, the oxidation signal is distorted, a behavior that does not occur with parameters of MA = 0.2 V, MT = 0.2 s, and TI = 0.2 s, these being the parameters chosen. Furthermore, these parameters yield a lower background current (see Figure S1a). On the other hand, different pre-concentration voltages were applied from −0.8 V to −1.1 V; it was found that, at higher potential values, the Cu(II) current intensity peak is more intense and defined down to −1.0 V, at −1.1 V a similar signal was obtained. Therefore, −1.0 V was chosen for the pre-concentration (see Figure S1b). Finally, the effect of the Cu pre-concentration time in a range from 20 s to 240 s was analyzed; little variation in the intensity of the peak was observed, so 60 s was selected because, at this time, it generated a better-defined signal (see Figure S1c).

In the case of Fe(III), 80 μg L^−1^ was used in previously established support electrolyte KNO_3_ 0.1 mol L^−1^/HNO_3_ 0.01 mol L^−1^, and a potential window from −1.35 to –0.8 V was obtained. In order to improve the Fe(III) response signal, the MA, MT, and TI parameters were varied from 0.05 V, 0.05 s, and 0.05 s to 0.3 V, 0.3 s, and 0.3 s, respectively. When the values of MA, MT, and TI were larger, a stronger and sharpest current signal was achieved; however, at values of MA = 0.3 V, MT = 0.3 s, and TI = 0.3 s; therefore, values of MT = 0.2 V, TM = 0.2 s and TI = 0.2 s were chosen. In addition, at these values, a lower background current is obtained (see Figure S2a). On the other hand, the pre-concentration potential was evaluated from −0.1 V to −1.8 V; at −1.6 V, the sharpest current signal was achieved (see Figure S2b). Finally, the study of the pre-concentration time of Fe(II) was carried out from 20 s to 180 s; little variation in the intensity of the peak was achieved, so 60 s was selected; in this time, the sharpest current signal was achieved (see Figure S2c).

#### 3.3.3. Calibration Curve, Limit of Detection, and Limit of Quantification in the Determination of Cd(II), Cu(II), and Fe(III)

Once the optimal parameters for the quantification of Cd(II), Cu(II), and Fe(III) were defined, the calibration plots were constructed using DPASV in BDD.

**Figure 6:**
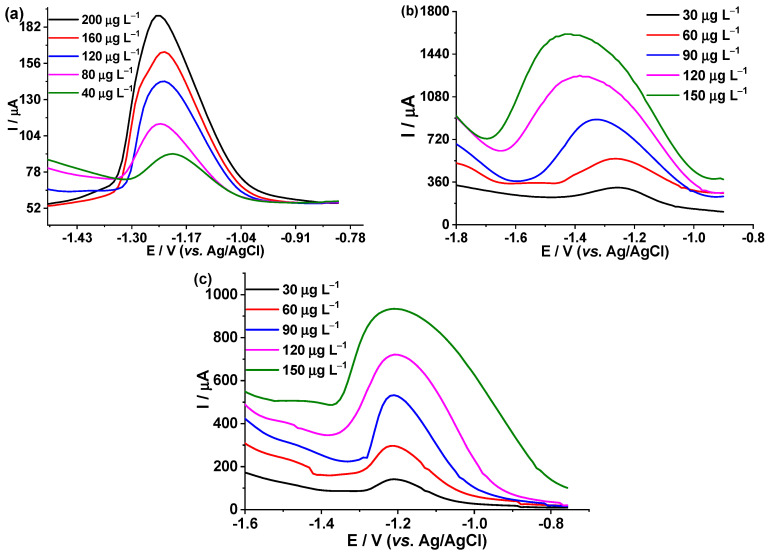
DPASV and calibration plots on the BDD electrode: (**a**) Cd(II) in support electrolyte, 0.1 mol L^−1^ acetic acid/sodium acetate at pH 4.5; (**b**) Cu(II) in electrolyte support of KNO_3_ 0.1 mol L^−1^/HNO_3_ 0.1 mol L^−1^; (**c**) Fe(III) in electrolyte support of KNO_3_ 0.1 mol L^−1^/HNO_3_ 0.01 mol L^−1^.

For Cd(II), we worked in concentrations of 40 to 200 μg L^−1^ in electrolyte support, 0.1 mol L^−1^ acetic acid/sodium acetate at pH 4.5, in the potential range from −1.4 V to −1.0 V, see Figure 6a. For Cu(II), we worked in concentrations of 30 to 150 μg L^−1^ in electrolyte support KNO_3_ 0.1 mol L^−1^/HNO_3_ 0.1 mol L^−1^, in the potential range of −1.7 V at −1.0 V, Figure 6b. In the case of Fe(III), we worked at concentrations of 30 to 150 μg L^−1^ of Fe(III) in a supporting electrolyte KNO_3_ 0.1 mol L^−1^/HNO_3_ 0.01 mol L^−1^, in the potential range of −1.4 to −0.8 V (see Figure 6c).

Figure S3a–c shows the calibration plots for Cd(II), Cu(II), and Fe(III) (respectively); they show a linear behavior for the three metals. Table 2 summarizes the parameters of the calibration plots. Once the calibration plots were obtained, the current intensities were measured by DPASV of 10 solutions of each supporting electrolyte: acetic acid 0.1 mol L^−1^/sodium acetate at pH 4.5, KNO_3_ 0.1 mol L^−1^/HNO_3_ 0.1 mol L^−1^, KNO_3_ 0.1 mol L^−1^/HNO_3_ 0.01 mol L^−1^. With the results obtained, the detection limits (DL) and quantification limits (QL) for Cd(II), Cu(II), and Fe(III) were determined, see Table 2.

#### 3.3.4. Optimal Parameters of Differential Pulse Adsorption Stripping Voltammetry for the Determination of Cd, Cu, and Fe with Food Matrix Effect

In the case of Cd, DPASV was applied to previously digested, fortified, and graduated craft beer samples with 200 μg L^−1^ of Cd(II) in the electrolyte acetic acid 0.1 mol L^−1^/sodium acetate 0.055 mol L^−1^ at pH 4.5, the signal was generated between −1.8 V to −1.0 V, with broader peaks than those generated without matrix effect (−1.4 V to −1.0 V) (see Figure 6a and Figure S5a). To improve the signal, the parameters MA, MT, and TI were modified with respect to the pre-selected ones without matrix effect. MA, MT, and TI were varied from 0.5 V, 0.5 s, and 0.5 s to 0.7 V, 0.7 s, and 0.7 s, respectively; in these last values, the best signal with effect was obtained. Matrix for Cd(II) quantification (see Figure S5a).

In the case of Cu, 90 μg L^−1^ of Cu(II) was used in 0.1 mol L^−1^ KNO_3_/0.1 mol L^−1^ HNO_3_ in previously digested craft beer samples. When applying DPASV, Cu(II) signals between −1.8 V to −0.7 V were obtained, in the same way, with broader peaks than those generated without matrix effect (−1.7 V to −1.0 V) (see Figure 6b and Figure S5b). To improve the signal, MA, MT, and TI were varied from 0.5 V, 0.5 s, and 0.5 s to 0.8 V, 0.8 s, and 0.8 s, respectively. When the values of MA, MT, and TI were larger, a stronger and more defined current signal was achieved. However, the parameters MA = 0.7 V, MT = 0.7 s, and TI = 0.7 s were chosen for the quantization of Cu since, with these parameters, there is a lower current of background (see Figure S5b).

Finally, in the case of Fe, 80 μg L^−1^ of Fe (III) was used in 0.1 mol L^−1^ KNO_3_/0.01 mol L^−1^ HNO_3_ in previously digested craft beer samples in which when applying DPASV. Fe(III) signal appeared between −1.95 to −0.80 V, with broader peaks than those generated without the matrix effect (−1.4 V to −0.8 V) (see Figure 6c and Figure S5c). To improve the signal, MA, MT, and TI were varied from 0.4 V, 0.4 s, and 0.4 s to 0.7 V, 0.7 s, and 0.7 s, respectively. When the values of MA, MT, and TI were larger, a stronger and more defined current signal was achieved, see Figure S2a. Therefore, these last parameters were selected as optimal for the quantification of Fe in the solutions prepared from craft beers (see Figure S5c).

#### 3.3.5. Standard Addition Plot of Cd(II), Cu(II), and Fe(III)

Having defined the optimal parameters for the electrochemical detection of Cd, Cu, and Fe in craft beer, the calibration plot was constructed in the concentration interval from 0 to 200 μg L^−1^ for Cd(II) (see Figure S5a) and in the interval from 0 to 150 μg L^−1^ for Cu(II) and Fe(III) (see Figure S5b,c), Table 3 summarizes the parameters of the calibration plots, with and without matrix effect. When comparing the calibration plots for Cd(II), Cu(II), and Fe(III), a higher slope is identified when there is a matrix effect, Table 3. The coefficient of determination varies slightly without and with the food matrix, Table 3. 

#### 3.3.6. Repeatability, Reproducibility, and Recovery in the Determination of Cd, Cu, and Fe

Once the calibration plots were established by standard addition, the repeatability, reproducibility, and percentage recovery of the analytes by DPASV were evaluated. Table 4 reports the percentage values of relative standard deviation (RSD%) in three days, repeatability, and reproducibility of the DPASV method expressed RSD% for each element.

**Figure 7:**
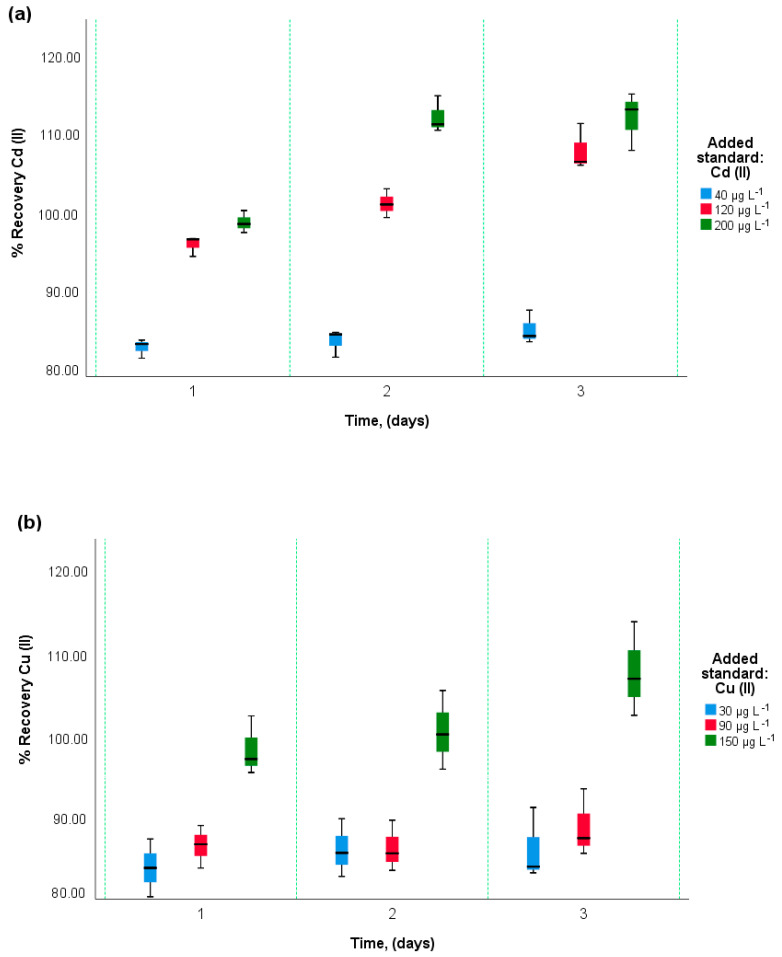

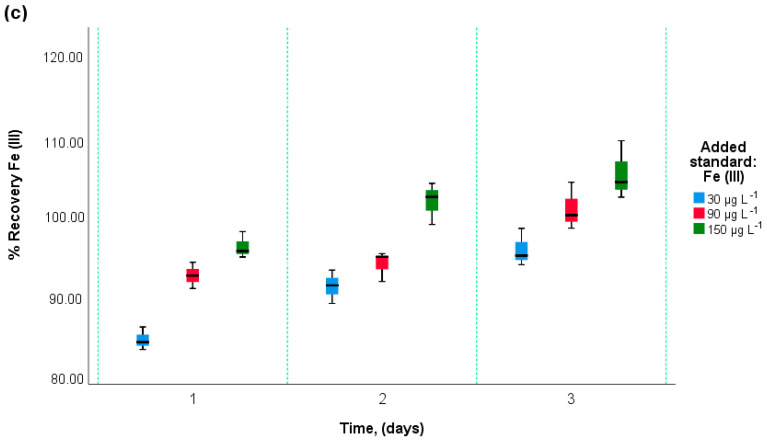
Box-and-whisker plot of percent recovery, per day, per added standard: (**a**) Cd(II) recovery; (**b**) Recovery of Cu(II); (**c**) Recovery of Fe(III).

In Figure 7a–c, box and whisker diagrams of the recovery percentage are shown, where it is observed that by increasing the concentration by standard addition of Cd(II), Cu(II), and Fe (III), there is a tendency to recover more of the analyte. In the interday precision of the measurements (3 days of evaluation), a higher percentage of recovery (R%) was obtained for the addition of 200 μg L^−1^ Cd(II), 150 μg L^−1^ Cu(II), and 150 μg L^−1^ Fe(III). While a lower recovery in the standard addition of 40 μg L^−1^ of Cd(II), 30 μg L^−1^ of Cu(II), and 30 μg L^−1^ of Fe(III). It can also be seen that the recovery tends to vary, increasing slightly as the days of repetition of the analysis progress. Globally, during the days of evaluation, the average R% of Cd(II), Cu(II), and Fe(III) was 94.94%, 91.68%, and 96.79%, respectively. The results indicate that the method for the determination of these three metals is accurate in time (3 days) since the values are within the acceptable range (80–120%) [52,53]. The recovery percentages obtained are similar to those reported for heavy metals by Tefela and Ayele (2020), 90.9 to 104.3% [54]. 

### 3.4. Results of Concentration of Cd, Cu, and Fe in Craft Beers

Table 5 shows the average concentrations of Cd, Cu, and Fe in craft beers obtained by DPASV. The results show some samples with the presence of metals that are not suitable are reported for human consumption because their metal concentrations exceed the limits allowed by the Ecuadorian norm NTE INEN 2263 (concentrations of iron and copper < 0.2 mg L^−1^, <1.0 mg L^−1^, respectively) [33]. Beer regulations and Brazilian legislation on heavy metals for fermented alcoholic beverages [34] establish cadmium concentrations < 0.5 mg L^−1^; the beers that do not comply with the regulations are CAR-A, CAR-E, CAR-H, CAR-H, CAR-K, and CAR-M. On the contrary, the craft beers CAR-B, CAR-C, CAR-D, CAR-F, CAR-G, CAR-I, CAR-J, and CAR-L, although they contain heavy metals, do not exceed the limits allowed by the reference regulations.

**Figure 8:**
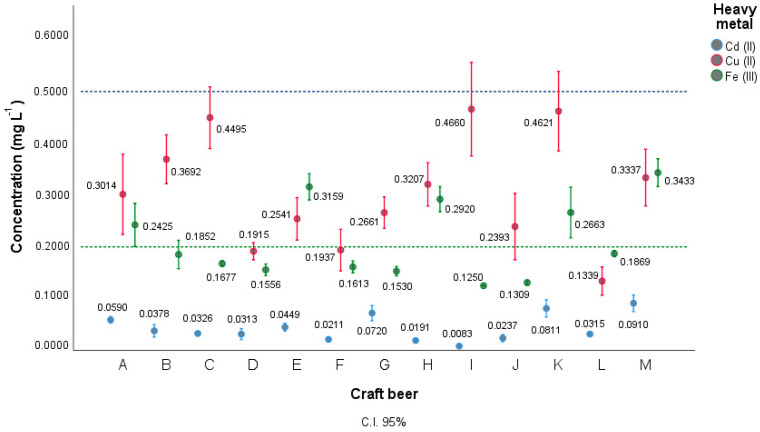
Plot of pooled confidence intervals of heavy metal concentration for craft beer.

Figure 8 represents a diagram of the average concentrations of Cd, Cu, and Fe for each craft beer, with 95% confidence intervals (CI), resolved in IBM SPSS Statistics. It can be highlighted that there is a higher concentration of Cu compared to the other metals analyzed, with the CAR-C, CAR-I, and CAR-K beers having a higher concentration (0.4495, 0.4660, and 0.4621 mg L^−1^, respectively) and greater variability of the results (error bar/CI 95%). On the contrary, the Cd analyzed represented the lowest concentration compared to the other metals, with the CAR-F, CAR-H, CAR-I, CAR-J, and CAR-L beers having the lowest concentration (0 0.0211, 0.0191, 0.0083, 0.0237 and 0.0315, mg L^−1^, respectively) and less variability of the results. Both the concentrations of Cd and Cu are very far from the reference line, which is why they are legally suitable for consumption, unlike Fe, where some samples exceed the reference line (0.2 mg L^−1^).

#### Comparison of the Results Obtained between DPASV and FAAS in the Determination of Cd, Cu, and Fe

Table 6 reports the average concentration values of Cd, Cu, and Fe in craft beers obtained by DPASV and FAAS. Cd concentrations vary between 0.0083–0.0910 mg L^−1^ by DPASV and 0.0137–0.0736 mg L^−1^ by FAAS. Cu concentrations vary between 0.1339–0.4660 mg L^−1^ and 0.0839–0.05175 mg L^−1^ for DPASV and FAAS, respectively; and, regarding Fe, it can be identified that the concentrations vary between 0.1250–0.3159 mg L^−1^, for the DPASV method, while for FAAS it was not quantified due to the low sensitivity of the method (QL = 0.3 mg L^−1^).

Student’s “*t*” statistical test between the DPASV and FAAS methods in determining Cd resulted in a statistical “*t*” of 2.080, a value that does not exceed the critical “*t*” of 2.18 for 12 degrees of freedom and for two tails. In the Student’s *t*-test between the DPASV and FAAS methods in the determination of Cu, the *t* statistic was 1.649, a value that also did not exceed the critical *t* of 2.18 for 12 degrees of freedom and for two tails; that is, in both cases (Cd and Cu), there is no statistically significant difference between methods.

## 4. Discussion

According to Figure 1, the BDD crystals are on the nanometric scale (300 to 2000 nm), which allows for a larger contact surface and it increases the number of active sites where electrochemical reactions can take place [55,56]. Proper cleaning allows residual carbon impurities to be removed from the BDD surface, which reduces possible interferences and increases the reproducibility of analytical signals. The low percentage of oxygen, close to 1%, determined by EDX, is related to the low amount of C-sp^2^ bonds, suitable for electroanalytical studies. In addition, in this study, 4.91 atomic % boron, 4.43 % by weight, was obtained by EDX, which confers conductivity to the diamond electrode.

The working potential window using cyclic voltammetry is the potential interval in which neither the electrolyte nor the solvent reacts. For the BDD electrode in KCl 1 mol L^−1^, the window was from −1.15 V to 1.60 V; this interval agrees with what has been reported in the literature [29,45,57].

Double layer capacitance (C_dl_) is the amount of charge that an electrode stores when it is polarized. When the C_dl_ has a high value, the capacitive current overlaps the faradaic current during the measurement, which is very detrimental when working with analytes at trace levels. The value obtained for the BBD electrode in this study was 0.01412 μF cm^−2^, relatively low according to what was reported by Kim et al. (2013) for the BDD electrode, 15.2 μF cm^−2^ [45,58]. The C_dl_ is directly linked to the amount of C-sp^2^, and with the boron doping level of the BDD, a higher concentration of C-sp^2^, a higher C_dl_ is expected [59]. It is furthermore considered that the low double layer electrical capacitance of BDD electrodes derives from the low density of electronic states (DOS) at the Fermi level [60].

One of the most widely used redox couples to characterize carbonaceous materials electrode is K_3_[Fe(CN)_6_]/K_4_[Fe(CN)_6_]. In the BDD electrode, this redox pair presents characteristic responses, depending on its surface termination; when the surface of BDD contains abundant oxygen (large amount of C-sp^2^), the redox process is slow and irreversible, ∆E takes large values, the opposite occurs when the surface is less oxygenated [61,62,63]. In the insert of Figure 4, the linear behavior of plot I vs. ν^1/2^ can be verified that the process is purely diffusional, and there is no adsorption or other adjacent phenomena, according to the Randles–Sevcik equation [64,65]. 

On the other hand, k° = 2.44 × 10^−2^ ± 4.67 × 10^−3^ cm s^−1^ was determined with the Nicholson equation [35], Equation (3) using an average value of ΔE de 96.74 mV; this value is similar to those obtained by Konkova et al. (2020), ΔE of 110 V [66]. These results indicate that the redox process is fast and quasi-reversible. It is important to indicate that when the ΔE of the redox system is close to 0.059 V, it is considered a fast and quasi-reversible redox process. On the contrary, if the ΔE is very large, it corresponds to a slow and irreversible process. According to Liu et al. (2023) and De Souza et al. (2019) [48,67], the recommended *v* to study kinetic parameters are from 20 mV s^−1^ to 100 mV s^−1^, since at *v* > 100 mV s^−1^, the capacitances substantially influence the shape of the voltammogram. On the contrary, at *v* < 20 mV s^−1^, the species formed diffuse into the bulk, and reversibility in the electrochemical response is lost when the reverse process is run [46,68].

When Ip_ox_/Ip_red_ is equal to 1, it is considered a fast and reversible redox process; when it is close to 1, it is considered a quasi-reversible redox process; and when it is very different from 1, the process is considered slightly reversible or irreversible [69]. In this study, Table S1, a value of Ip_ox_/Ip_red_ = 0.99 was obtained; that is, the redox process for the potassium ferro-ferricyanide system in BDD is quasi-reversible. 

One of the key variables in electroanalysis to achieve a well-defined and sharpest current signal is the electrolyte used and the pH. In this study, the best signals were achieved using 0.1 mol L^−1^ acetic acid/0.055 mol L^−1^ sodium acetate at pH 4.5; KCl 0.1 mol L^−1^/HCl 0.01 mol L^−1^ and KNO_3_ 0.1 mol L^−1^/HNO_3_ 0.01 mol L^−1^ to quantify Cd(II), Cu(II) and, Fe(III) by DPASV, respectively. The reaction medium directly influences the solvation sphere of the analyte and its activation energy when electrochemically inducing a transformation [30,50,51].

It was evidenced that the response signals do not grow symmetrically when there is an effect of the food matrix; this is due to the presence of analytical interference typical of the matrix [70]. 

The DL and QL for Cd(II), Cu(II), and Fe(III) by means of DPASV in the BDD electrode are low values and adequate for chemical elements in low concentrations, such as in craft beers. On the other hand, the DL and QL are lower for Fe(III) (DL = 1.72, QL = 5.72, μg L^−1^) compared to Cu(II) (DL = 1.76, QL = 5.87, μg L^−1^) and much lower with respect to Cd(II) (DL = 6.31, QL = 21.04, μg L^−1^). The DL of Cu and Fe are relatively lower than those reported by Passaghe (2015) [71] and Bonilla (2022) [72] (4.12 μg L^−1^ and 36.0 μg L^−1^, respectively); while the DL of Cd is relatively higher than that reported by Marcano (2010) [73] (1.8 μg L^−1^), however, this reflects that the method is acceptable.

Despite not obtaining Fe values in craft beers by FAAS, the results obtained for Fe by DPASV show that there is a presence of the metal, indicating greater sensitivity of the DPASV method, and confirm, in addition, that the electrochemical method is appropriate to detect elements in very low concentrations. 

The DPASV method applied on the BDD electrode, having RSD% below 10% and recoveries above 90%, therefore, has acceptable precision and accuracy, respectively, for the quantification of these heavy metals in the ionic state [74].

The concentrations of Cd reported in this study (cervezas de Quito, Ecuador) (0.0083–0.0910 mg L^−1^ of Cd(II)) are lower than the results of the study by Becerra (2014) [26] in craft beers from Cuenca-Ecuador, where values higher than 0.1 mg L^−1^ were found for Cd in blonde type beer. The concentrations of Cu (0.1339–0.4660 mg L^−1^ of Cu) were also in a lower range compared to those of Becerra, where concentrations greater than 17 mg L^−1^ Cu were found in black type. Regarding the Fe content (0.1250–0.3159 mg L^−1^) from this study, it was lower than that reported by Zambrzycka-Szelewa et al. (2020) [75] in craft beers from Bialystok-Poland, range of 0.0624–1.199 mg L^−1^. As can be seen, the concentration of heavy metals is variable depending on the origin, type of beer, or the way in which they were made during production. 

From this study, five beers have a concentration above the permissible limits of the food regulation NTE INEN 2262 [33] (0.2 mg L^−1^), so the consumption of these drinks is not accepted. Since iron is an essential element of the human diet, the intake between 10–20 mg day^−1^ [76,77] is recommended; however, it should not exceed 20 mg day^−1^ since it can cause damage to health, as stated by Ho Wang (2022) [78]. 

In the evaluated beers, higher concentrations of heavy metals were found in those with a high degree of alcohol; in addition, it was possible to demonstrate a high Fe(III) content in the CAR-A, CAR-E, CAR-H, CAR-K, and CAR M beers and that within their brewing ingredients, they contained caramelized barley malt, and sometimes roasted barley (CAR-A, CAR-E, CAR-H). The extract from roasted barley involves applying hot water favoring the solubility of the metal. In this sense, it can be corroborated that the ingredients used for the elaboration of the craft beer give the presence and increase of heavy metals. In addition, in an aqueous medium, the high alcoholic degree leads to certain biomolecules of the food matrix dissolving, releasing together with them the metal in an ionic state (Sancho, 2010). Sancho (2010) describes that the concentrations of free iron were lower for those that did not contain alcohol, and the composition of ingredients such as roasted barley influenced the concentration of the metal in the dark beers in their study [28].

## 5. Conclusions

The DPASV electrochemical method using BDD as the working electrode allowed the evaluation of Cd(II), Cu(II), and Fe(III) in craft beers. The selection of supporting electrolytes, pH range, and voltage modulation parameters play an important role in obtaining a defined signal for metal quantification. The DPASV method applied on the BDD electrode has acceptable precision and accuracy for the quantification of these heavy metals in the ionic state, which corroborates its good repeatability and reproducibility of the method used compared to other electrochemical studies [27,28,79]. The thirteen Quito craft beers have amounts of Cd (II), Cu (II), and Fe (III) that in most brands tend to be low concentration values; the concentration ranges were 0.0083–0.0910 mg L^−1^ of Cd(II), 0.1339–0.4660 mg L^−1^ of Cu(II) and 0.1250–0.3159 mg L^−1^ of Fe(III). On the other hand, the content of Cd and Cu quantified in the 13 craft beers does not represent a risk to the health of consumers. However, it was verified that some of the beers did not comply with the permissible limits of the NTE INEN 2262 standards. Five of the thirteen brands were outside the acceptable concentration limit in Fe(III); these beers were those coded as CAR-A, CAR-E, CAR-H, CAR-K, and CAR-M, which are red and that are sold in both the central north and north sectors of the city of Quito, which are not considered suitable for human consumption.

## Figures and Tables

**Table 1:** Identification of craft beer samples.

Beer Code	Origin	Sector of Sale ^a^	Specific Ingredients ^b^	Characteristic ^c^
CAR-A	Germany	Diego de Almagro	Caramel barley malt, roasted barley, brown sugar	IPA, 7.0 Vol%
CAR-B	Ecuador	Foch	Caramel barley malt	Ale, 5.0 Vol%
CAR-C	Ecuador	Avenue 12 de Octubre	Honey bee	Ale, 6.5 Vol%
CAR-D	Scotland	Pradera	Roasted barley, roasted hazelnuts	Ale, 6.0 Vol%
CAR-E	Ecuador	Avenue Orellana	Caramel barley malt, roasted barley,	Ale, 7.0 Vol%
CAR-F	Ecuador	Río Coca	Natural blackberry flavoring	Ale, 6.0 Vol%
CAR-G	Ireland	Avenue Oswaldo Guayasamin	Caramel barley malt, roasted barley	Ale, 7.0 Vol%
CAR-H	Ecuador	Avenue Whymper	Caramelized malt, roasted barley	Ale, 5.7Vol%
CAR-I	Ecuador	Avenue Mariana de Jesús	Caramel barley malt	Ale, 6.0 Vol%
CAR-J	Ecuador	Avenue 6 de Diciembre	Cinnamon, vanilla	Ale, 5.0 Vol%
CAR-K	Ecuador	Avenue Vaca de Castro	Caramelized malt, Blackberry, cocoa, cinnamon	Ale, 7.0 Vol%
CAR-L	Ecuador	Bicentenario		Ale, 5.0 Vol%
CAR-M	Ecuador	Reina Victoria	Caramelized malt, Cotton candy flavoring	Ale, 6.9 Vol%

^a^ In the city of Quito. ^b^ Common ingredients: water, malt, hops, and yeast, omitted for clarity. ^c^ All beers are red type.

**Table 2:** Parameters of the calibration curves of the 3 metals.

Metal	Slope of the Curve μA (μg/L)^−1^	Correlation Coefficient, r	Determination Coefficient, R^2^	DL μg L^−1^	QL μg L^−1^
Cd(II)	0.62665	0.999	0.998	6.31	21.04
Cu(II)	10.9618	0.998	0.994	1.76	5.87
Fe(III)	6.71163	0.999	0.997	1.72	5.72

**Table 3:** Parameters of the calibration curves of the 3 metals with and without matrix effect.

Metal	Slope of the Plot μA (μg/L)^−1^		Correlation Coefficient, r		Determination Coefficient, R^2^	
	No Matrix Effect	With Matrix Effect	No Matrix Effect	With Matrix Effect	No Matrix Effect	With Matrix Effect
Cd(II)	0.62665	10.004	0.999	0.994	0.98	0.984
Cu(II)	10.9618	59.7068	0.998	0.997	0.994	0.991
Fe(III)	6.71163	29.7239	0.999	0.993	0.997	0.984

**Table 4:** Repeatability and reproducibility of the DPASV method for the determination of Cd, Cu, and Fe.

Heavy Metal	Days	(RSD%)	Repeatability (RSD%)	Reproducibility (RSD%)
Cd(II)	1	1.06	1.06	1.61
	2	1.52		
	3	2.23		
Cu(II)	1	2.43	2.43	2.94
	2	2.89		
	3	3.49		
Fe(III)	1	1.34	1.34	1.83
	2	1.79		
	3	2.37		

**Table 5:** Concentration of Cd, Cu, and Fe in craft beers by DPASV.

Code of Beer	Heavy Metal	Average (mg L^−1^)	S_d_	RSD%	Complies with Food Standards *
CAR-A	Cd	0.0589	0.0262	4.44	Yes
	Cu	0.3015	0.0312	10.36	Yes
	Fe	0.2425	0.0168	6.92	No
CAR-B	Cd	0.0378	0.0496	13.12	Yes
	Cu	0.3692	0.0190	5.15	Yes
	Fe	0.1851	0.0111	5.98	Yes
CAR-C	Cd	0.0326	0.0010	3.10	Yes
	Cu	0.4495	0.0240	5.34	Yes
	Fe	0.1677	0.0024	1.41	Yes
CAR-D	Cd	0.0313	0.0431	13.75	Yes
	Cu	0.1915	0.0067	3.50	Yes
	Fe	0.1556	0.0046	2.93	Yes
CAR-E	Cd	0.0449	0.0297	6.62	Yes
	Cu	0.2541	0.0165	6.51	Yes
	Fe	0.3159	0.0102	3.22	No
CAR-F	Cd	0.0211	0.0178	8.42	Yes
	Cu	0.1937	0.0162	8.34	Yes
	Fe	0.1613	0.0047	2.88	Yes
CAR-G	Cd	0.0720	0.0587	8.15	Yes
	Cu	0.2661	0.0123	4.61	Yes
	Fe	0.1530	0.0037	2.44	Yes
CAR-H	Cd	0.0191	0.0064	3.36	Yes
	Cu	0.3207	0.0168	5.25	Yes
	Fe	0.2920	0.0098	3.35	No
CAR-I	Cd	0.0083	0.0096	11.64	Yes
	Cu	0.4660	0.0364	7.82	Yes
	Fe	0.1250	0.0003	0.21	Yes
CAR-J	Cd	0.237	0.0286	12.07	Yes
	Cu	0.2393	0.0259	10.83	Yes
	Fe	0.1309	0.0018	1.34	Yes
CAR-K	Cd	0.0811	0.0676	8.34	Yes
	Cu	0.4621	0.0310	6.71	Yes
	Fe	0.2663	0.0197	7.39	No
CAR-L	Cd	0.0315	0.0010	3.20	Yes
	Cu	0.1339	0.0110	8.20	Yes
	Fe	0.1869	0.0024	1.30	Yes
CAR-M	Cd	0.0910	0.0651	7.15	Yes
	Cu	0.3337	0.0221	6.61	Yes
	Fe	0.3433	0.0109	3.17	No

* According to Ecuadorian regulations NTE INEN 2263, corresponding to alcoholic beverages and Beer Regulations and Brazilian legislation on heavy metals for fermented alcoholic beverages. These regulations establish the amounts of iron, copper, and cadmium allowed: <0.2 mg L^−1^, <1.0 mg L^−1^ [33], and <0.5 mg L^−1^ [34], respectively.

**Table 6:** Comparison of Cd content obtained by DPASV and FAAS.

Beer Code	Cd		Cu		Fe	
	DPASV (mg L^−1^)	FAAS (mg L^−1^)	DPASV (mg L^−1^)	FAAS (mg L^−1^)	DPASV (mg L^−1^)	FAAS (mg L^−1^)
CAR-A	0.0589	0.0335	0.3015	0.3931	0.2425	ND *
CAR-B	0.0378	0.0281	0.3692	0.2264	0.1851	ND
CAR-C	0.0326	0.0366	0.4495	0.3732	0.1677	ND
CAR-D	0.0313	0.0270	0.1915	0.1156	0.1556	ND
CAR-E	0.0449	0.0320	0.2541	0.2837	0.3159	ND
CAR-F	0.0211	0.0233	0.1937	0.2153	0.1613	ND
CAR-G	0.0720	0.0553	0.2661	0.1354	0.1530	ND
CAR-H	0.0191	0.0224	0.3207	0.2860	0.2920	ND
CAR-I	0.0083	0.0137	0.4660	0.3947	0.1250	ND
CAR-J	0.0237	0.0236	0.2393	0.1764	0.1309	ND
CAR-K	0.0811	0.0664	0.4621	0.5175	0.2663	ND
CAR-L	0.0315	0.0384	0.1339	0.0839	0.1869	ND
CAR-M	0.0910	0.0736	0.3337	0.3555	0.3770	ND

* ND: Not detected.

## Data Availability

The thesis has not yet been published. The results obtained are available in this manuscript.

## Supplementary Materials

The following supporting information can be downloaded at: https://www.mdpi.com/article/10.3390/foods12112264/s1.
