# Supplementary material for: Electrochemical Evaluation of Cd, Cu, and Fe in Different Brands of Craft Beers from Quito, Ecuador
Source: Foods. 2023 Jun 4;12(11):2264. doi: 10.3390/foods12112264 (PMC10252223; doi:10.3390/foods12112264)
Supplement: Supplementary file 1 [file foods-12-02264-s001.zip › foods-2323228-supplementary.pdf]

Soportes

Tables

Table S-1. Cyclic voltammetry of $K_4[Fe(CN)_6]$ / $K_3[Fe(CN)_6]$ on BDD electrode.

υ ($V\ s^{-1}$)	ΔEp (mV)	$Ep_{1/2}$ (mV)	Ip_{ox} (μA)	Ip_{red} (μA)	Ip_{ox}/Ip_{red}	k° ($cm\ s^{-1}$)
0.020	74.78	372.90	193.2412	195.8914	0.99	3.25×10^{-02}
0.040	87.86	372.77	275.0711	278.4573	0.99	2.70×10^{-02}
0.060	96.10	371.78	335.5251	339.4322	0.99	2.41×10^{-02}
0.080	101.09	371.84	385.0713	388.0118	0.99	2.20×10^{-02}
0.100	107.50	371.63	430.0244	432.3939	0.99	2.09×10^{-02}
0.120	113.04	372.27	468.9341	471.5781	0.99	2.01×10^{-02}
Average						$2.44 \times 10^{-2} \pm 4.67 \times 10^{-3}$

Figures

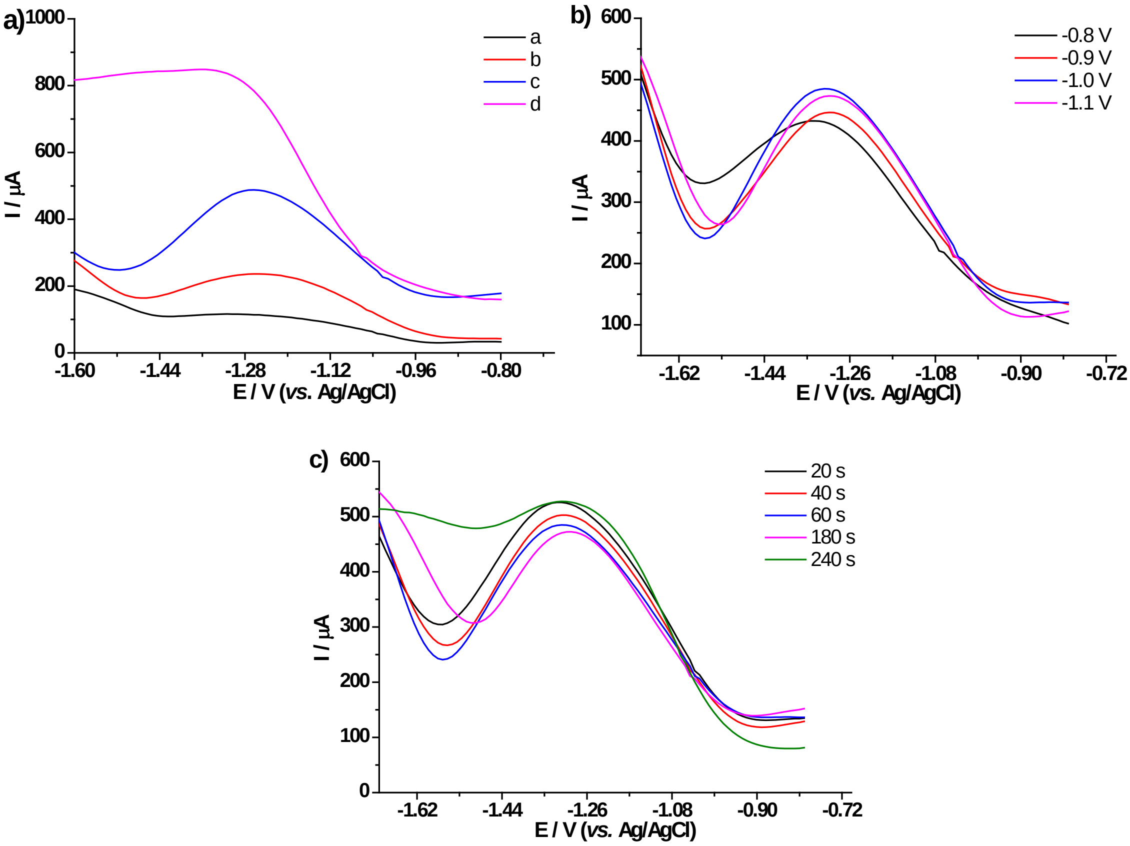

Figure S-1. Dissolution behavior of 90 μg L^{-1} of Cu(II) in electrolyte KNO_3 0.1 mol L^{-1} / HNO_3 0.1 mol L^{-1} by DPASV at different parameters: **(a)** from MA 0.05 V, MT 0.05 s, TI 0.05 s - MA 0.3 V, MT 0.3 s, TI 0.3 s. **(b)** from -0.8 V to -1.1 V. **(c)** from 20 s to 240 s.

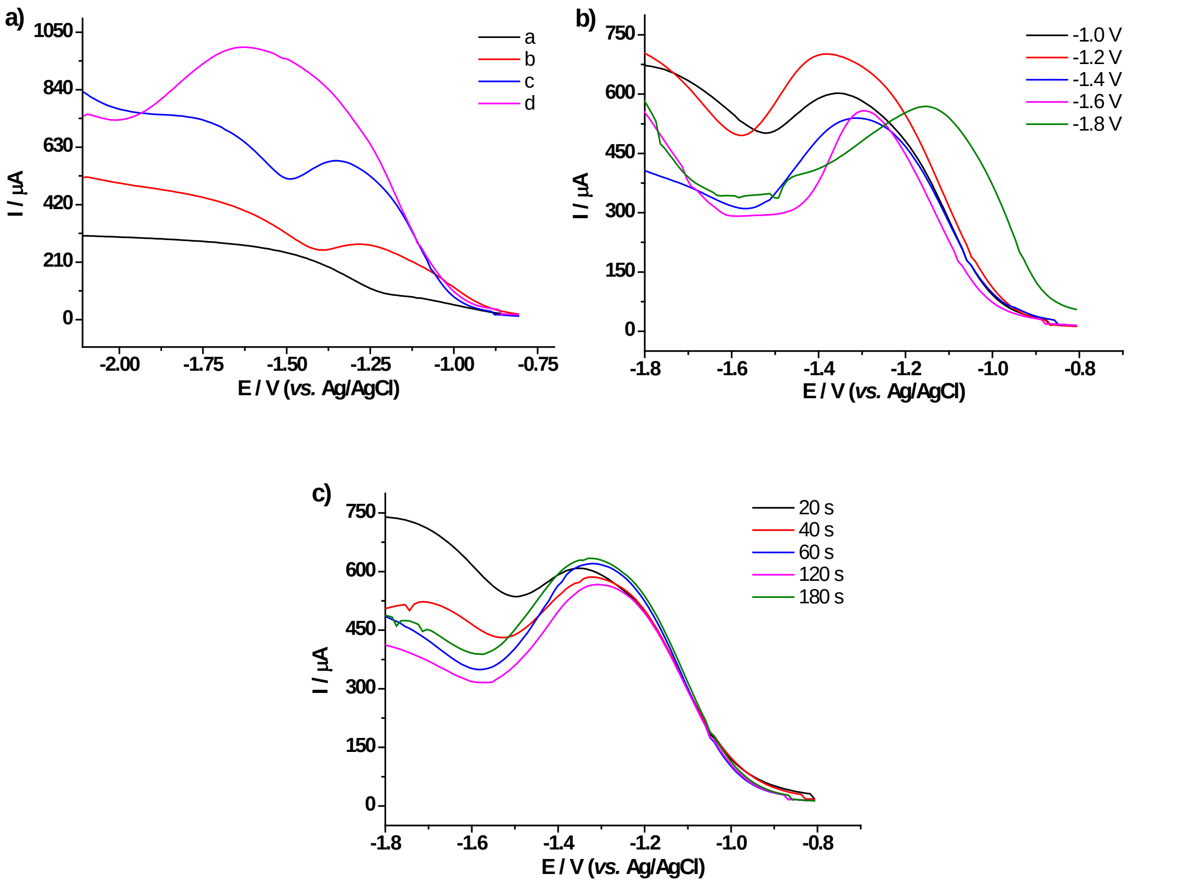

Figure S-2. Dissolution behavior of 80 µg L^{-1} of Fe(III) in KNO_3 0.1 mol L^{-1} / HNO_3 0.01 mol L^{-1} electrolyte at different DPASV parameters: **(a)** from MA 0.05 V, MT 0.05 s, TI 0.05 s - MA 0.3 V, MT 0.3 s, TI 0.3 s. **(b)** from -1.0 V to -1.8 V. **(c)** from 20 s to 180 s.

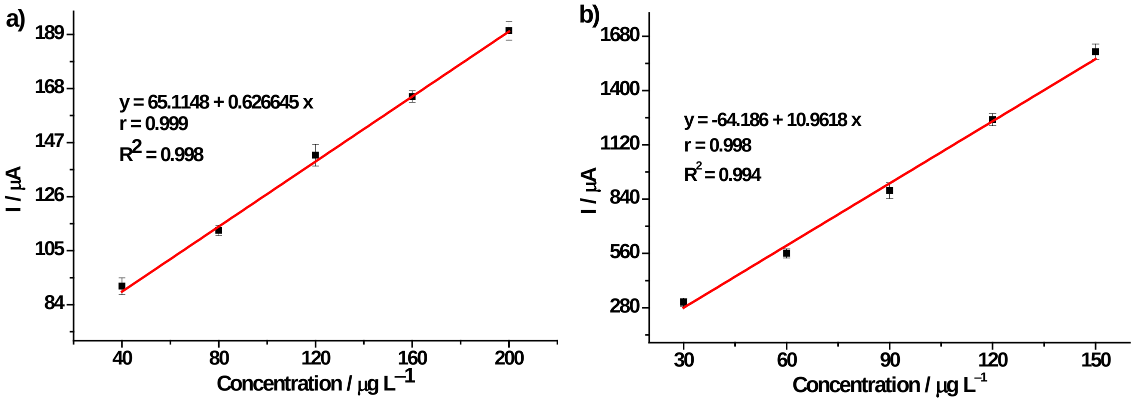

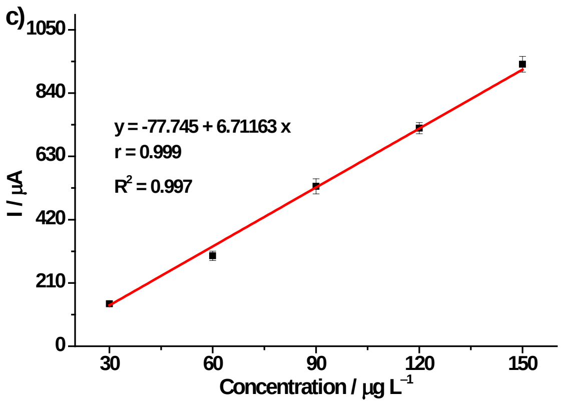

Figure S-3. Calibration plots of: **(a)** Cd(II); **(b)** Cu(II); **(c)** Fe(III).

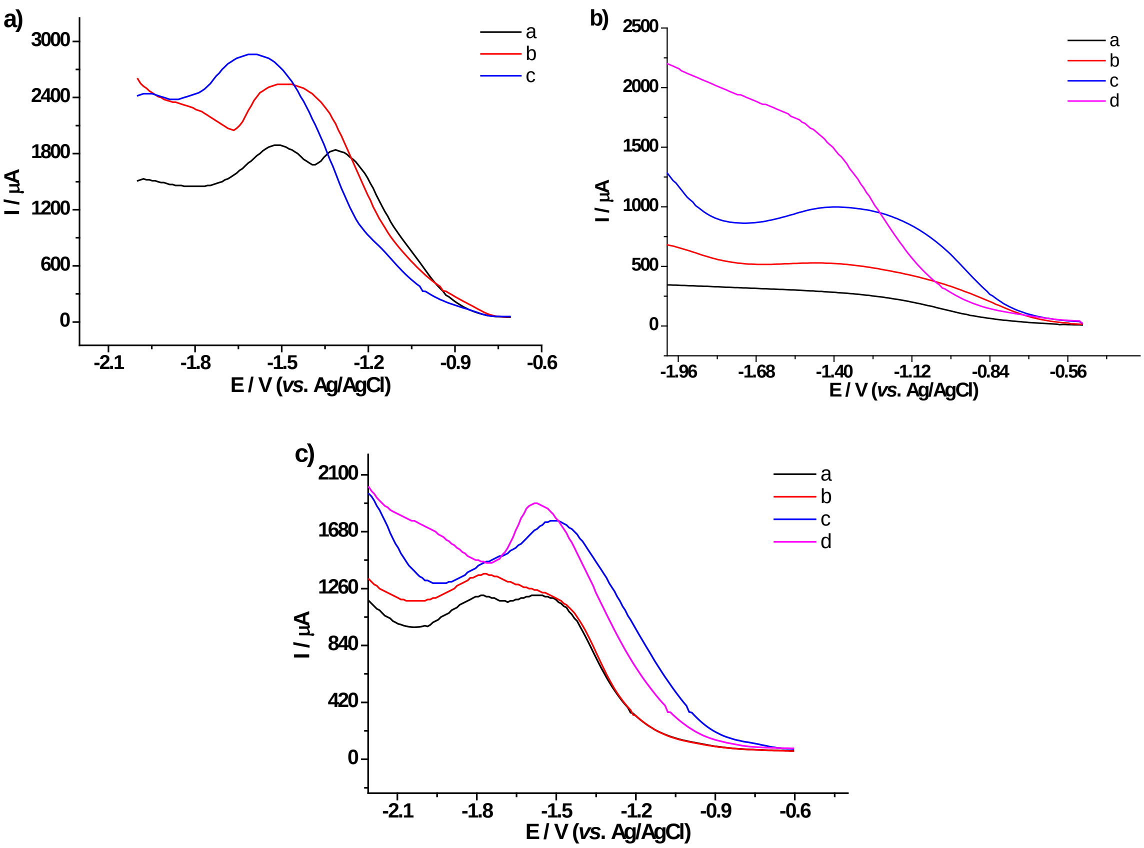

Figure S-4. Beer matrix dissolution behavior with metal standard: **(a)** 200 μg L^{-1} standard of Cd(II) in 0.1 mol L^{-1} acetic acid electrolyte / sodium acetate at pH 4.5, by DPASV at different parameters: a) MA 0.5 V, MT 0.5 s, TI 0.5 s; b) MA 0.6 V, MT 0.6 s, TI 0.6 s; c) MA 0.7 V, MT 0.7 s, TI 0.7 s. **(b)** standard of 90 μg L^{-1} of Cu(II) in electrolyte KNO_3 0.1 mol L^{-1} / HNO_3 0.1 mol L^{-1} by DPASV at different parameters: a) MA 0.5 V, MT 0 0.5 s, TI 0.5 s; b) MA 0.6 V, MT 0.6 s, TI 0.6 s; c) MA 0.7 V, MT 0.7 s, TI 0.7 s; d) MA 0.8 V, MT 0.8 s, TI 0.8 s. **(c)** 80 μg L^{-1} Fe(III) standard in 0.1 mol L^{-1} KNO_3 / 0.01 mol L^{-1} HNO_3 electrolyte at different DPASV parameters: a) MA 0.4 V, MT 0.4s, TI 0.4s; b) MA 0.5 V, MT 0.5 s, TI 0.5 s; c) MA 0.6 V, MT 0.6 s, TI 0.6 s; d) MA 0.7 V, MT 0.7 s, TI 0.7 s.

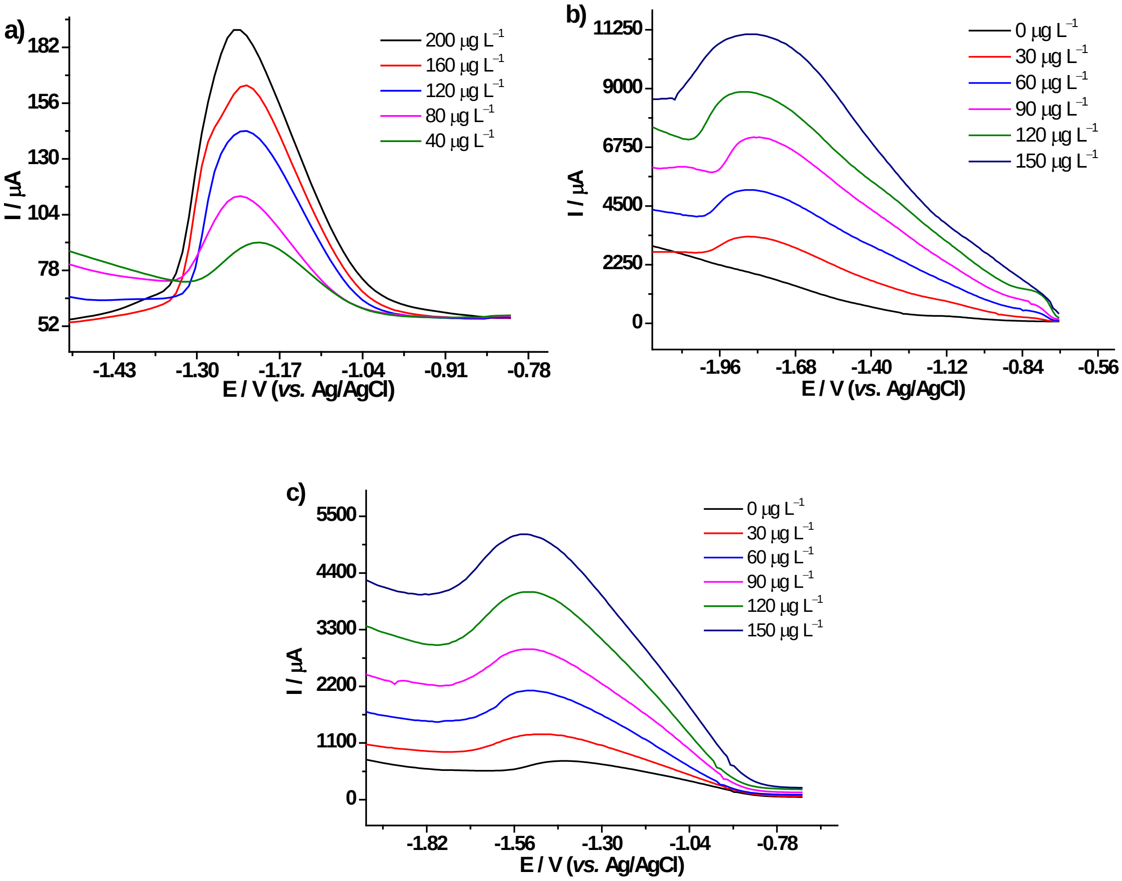

Figure S-5. Voltammogram and standard addition curve by DPASV in craft beer at different concentrations: **(a)** Cd(II); **(b)** Cu(II); **(c)** Fe(III).

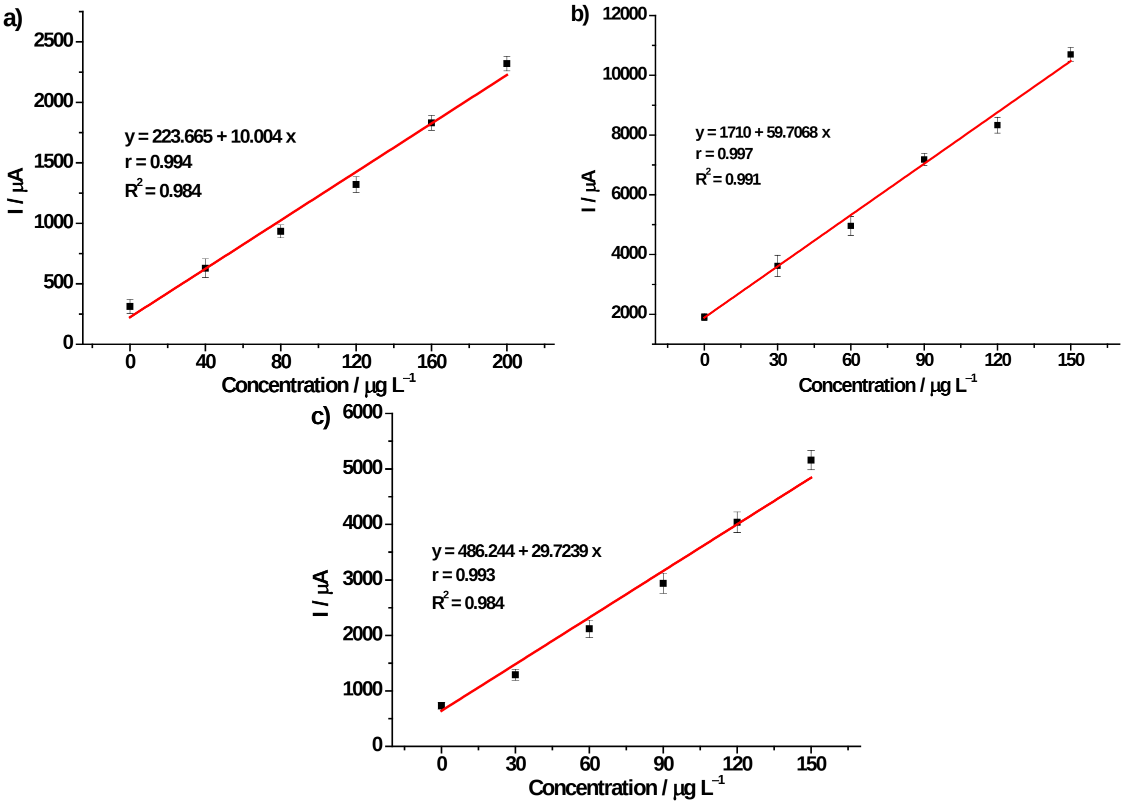

Figure S-6. Standard improvement calibration curves: (a) for Cd(II), (b) for Cu(II) and (c) for Fe(III).
